# Self‐Sacrifice Template Construction of Uniform Yolk–Shell ZnS@C for Superior Alkali‐Ion Storage

**DOI:** 10.1002/advs.202200247

**Published:** 2022-03-15

**Authors:** Xijun Xu, Fangkun Li, Dechao Zhang, Zhengbo Liu, Shiyong Zuo, Zhiyuan Zeng, Jun Liu

**Affiliations:** ^1^ School of Chemistry and Chemical Engineering and School of Materials Science and Engineering Guangdong Provincial Key Laboratory of Advanced Energy Storage Materials South China University of Technology Guangzhou 510641 China; ^2^ Department of Materials Science and Engineering City University of Hong Kong Hong Kong 999077 China

**Keywords:** alkali‐ion batteries, anode, long‐cycle life, yolk–shell, ZnS@C nanorods

## Abstract

Secondary batteries have been widespread in the daily life causing an ever‐growing demand for long‐cycle lifespan and high‐energy alkali‐ion batteries. As an essential constituent part, electrode materials with superior electrochemical properties play a vital role in the battery systems. Here, an outstanding electrode of yolk–shell ZnS@C nanorods is developed, introducing considerable void space via a self‐sacrificial template method. Such carbon encapsulated nanorods moderate integral electronic conductivity, thus ensuring rapid alkali‐ions/electrons transporting. Furthermore, the porous structure of these nanorods endows enough void space to mitigate volume stress caused by the insertion/extraction of alkali‐ions. Due to the unique structure, these yolk–shell ZnS@C nanorods achieve superior rate performance and cycling performance (740 mAh g^−1^ at 1.0 A g^−1^ after 540 cycles) for lithium‐ion batteries. As a potassium‐ion batteries anode, they achieve an ultra‐long lifespan delivering 211.1 mAh g^−1^ at 1.0 A g^−1^ after 5700 cycles. The kinetic analysis reveals that these ZnS@C nanorods with considerable pseudocapacitive contribution benefit the fast lithiation/delithiation. Detailed transmission electron microscopy (TEM) and X‐ray diffraction (XRD) analyses indicate that such yolk–shell ZnS@C anode is a typical reversible conversion reaction mechanism accomplished by alloying processes. This rational design strategy opens a window for the development of superior energy storage materials.

## Graphical abstract

Porous yolk–shell ZnS@C nanorods are fabricated via a hydrothermal method accompanied with an in situ dissolving and sulfuration route. Due to the unique yolk–shell design, this ZnS@C anode can effectually ameliorate the volume fluctuation, shorten the diffusion distance of alkali‐ions, and thus facilitate the electrochemical reaction kinetics, thereby earning excellent rate performance and ultra‐long cycling capability.

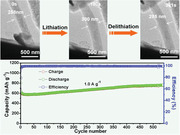

## 1 Introduction

Li‐ion batteries (LIBs) are the current popular power storage and conversion technologies for portable electronic devices, electric vehicles, laptops, and smart grids owing to their considerable energy density and high working voltage.^[^ ^1^, ^2^, ^3^ ^]^ Nevertheless, the limited lithium salt on the earth's crust seriously influences the widespread application of LIBs, especially for large‐scale energy storage stations. Recently, some similar and alternative battery systems based on alkali elements, such as potassium ion batteries (KIBs) and sodium‐ion batteries (SIBs) have attracted widespread concern because of their natural abundance of raw materials and low cost.^[^ ^4^, ^5^, ^6^ ^]^ Despite these advantages, the SIBs and KIBs with a radius of 1.02 Å and 1.38 Å, respectively, which is both larger than that of LIBs (0.76 Å).^[^ ^7^ ^]^ It means that design electrode materials are essential with large pathways and stable structures for Na^+^ and K^+^ migration. Therefore, there is still a bottleneck to explore appropriate electrode suitable for LIBs, SIBs, and KIBs. During the past few decades, various insertion‐type materials (e.g., graphite and Li_4_Ti_5_O_12_), conversion‐type materials (e.g., transition metal phosphides, selenides, sulfides, and oxides), and alloy‐type materials (e.g., Sn, Sb, and Bi) have been explored as anodes for LIBs.^[^ ^7^, ^8^, ^9^, ^10^, ^11^, ^12^, ^13^, ^14^, ^15^, ^16^, ^17^, ^18^, ^19^, ^20^, ^21^, ^22^ ^]^ Among them, insertion‐type materials possess a low theoretical capacity and volume capacity, and alloy‐type materials undergo large volume variation during charge/discharge leading to structural collapse and subsequent rapid capacity decay.^[^ ^17^, ^21^, ^22^ ^]^ Especially, conversion‐type materials with higher theoretical capacity than insertion‐type materials and relatively smaller volume fluctuation than alloy‐type materials have been focused on extensive attention.^[^ ^23^, ^24^ ^]^ Nevertheless, their practical application is still hampered by the aggregation of active metals and serious voltage hysteresis during the repeated cycles and thus resulting in unstable cycling performance and awful rate capability.^[^ ^25^, ^26^ ^]^ Moreover, most of the transition metal chalcogenides with high voltage platforms over 1.0 V will significantly decrease the total energy density of full batteries.^[^ ^27^, ^28^, ^29^, ^30^, ^31^, ^32^, ^33^ ^]^ Fortunately, zinc‐based compounds (e.g., ZnS and ZnSe) with low voltage platforms and abundant raw sources have attracted the researcher's attention.^[^ ^22^ ^]^ Recently, various efforts have been applied to the design of Zn‐based chalcogenides for alkali‐ion batteries. Prominently, a polyhedral carbon framework with the embedded ZnS nanoparticles designed by Sun's group displayed 840 mAh g^−1^ after 300 cycles at 0.6 A g^−1^ for LIBs.^[^ ^34^ ^]^ In the work of Pan's group, a polyhedral ZnS@C was achieved by a MOF‐derived strategy and acquired 370.6 mAh g^−1^ over 100 cycles at 0.1 A g^−1^ as SIBs anode.^[^ ^35^ ^]^ Bao's group designed double carbon modified ZnS@C@RGO anode, which enhanced electronic conductivity and achieved a stable specific capacity of 208 mAh g^−1^ over 300 cycles at 0.5 A g^−1^.^[^ ^36^ ^]^ Despite great efforts achieved in ZnS materials, the development of novel materials with a high capacity, long cycling lifespan, and superior rate capability is still with extensive opportunities in a surge of fields like SIBs and KIBs.^[^ ^37^, ^38^ ^]^ The nano/microstructure designing and carbon coating/encapsulation can effectively ameliorate the volume variation of active materials and preserve the integrity of multi‐structured electrodes from collapsing during the cycling process and thus improving the electrochemical performance. Generally speaking, the pyrolysis of the polymerized nitrogenous organic compounds could simply induce N‐doped carbon with in situ inherited active sites and defects, which could fantastically enhance the intrinsic electrochemical proprieties of electrodes.^[^ ^22^ ^]^ On account of ZnS‐based electrode materials, they could be facilely obtained by tuning of nano/microstructures of Zn‐based binary oxides.^[^ ^39^, ^40^ ^]^ Herein, organic polydopamine (PDA) is adopted for uniform surface coating of easily‐achieved Zn_2_GeO_4_ precursor nanorods. After a simple pyrolysis, the PDA coating shell transforms into N‐doped carbon, resulting in core‐shell Zn_2_GeO_4_@C nanorods. Then well‐defined yolk–shell ZnS@C is achieved by using Zn_2_GeO_4_@C as the self‐sacrifice template via hydrothermal sulfidation and selectively dissolving the Ge source at the same time. Such porous ZnS@C yolk–shell nanorods have abundant voids and defects, promising a long lifespan and superior rate capability for KIBs.

## 2 Results and Discussion

**Figure 1:**
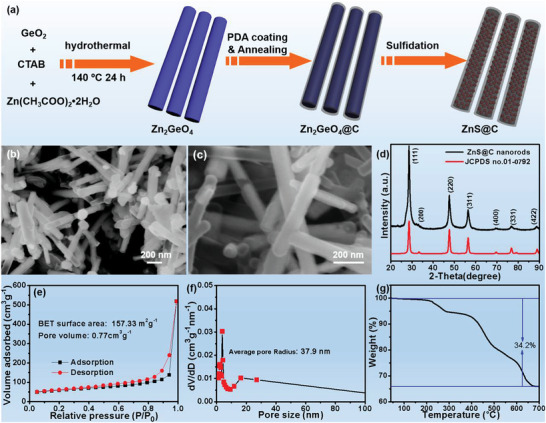
a) Schematic illustration of the yolk–shell ZnS@C nanorod formation; b,c) different magnification SEM images of ZnS@C; (d) XRD pattern of ZnS@C nanorods; nitrogen desorption/adsorption isotherms (e) and pore size distribution (f) of ZnS@C nanorods; g) TGA curve of ZnS@C nanorods.

The detailed fabrication procedure is illustrated in **Figure** **1**a, in which first, the Zn_2_GeO_4_ nanorods were achieved via a conventional hydrothermal strategy using the hexadecyl trimethyl ammonium bromide (CTAB) as surfactant. After that, these Zn_2_GeO_4_ nanorods were dully encapsulated by an organic PDA layer through a facile solution polymerization route and followed by annealing in an Ar flowing tube furnace to obtain Zn_2_GeO_4_@C. Finally, the yolk–shell ZnS@NC was obtained by selective dissolving and sulfidation of Zn_2_GeO_4_@C. As displayed in Figure S1, Supporting Information the prepared Zn_2_GeO_4_ possesses a nanorod structure with a diameter of ≈120 nm and a length of several micrometers. The X‐ray diffraction (XRD) pattern (Figure S2, Supporting Information) further reveals these Zn_2_GeO_4_ nanorods with a hexagonal phase. The finally scanning electron microscopy (SEM) images of ZnS@C reveal a nanorod structure (Figure 1b,c), which is well‐matched with the Zn_2_GeO_4_ precursor, strongly proving the feasibility of this unique design strategy. Figure 1d shows the detailed phase of the finally carbon‐coated product, confirming the successful synthesis of ZnS@C. Obviously, all mainly peaks of ZnS@C nanorods can be indexed to the (111), (220), (311), (400), (331), and (422) crystalline planes of cubic ZnS. Furthermore, the crystalline structures of Zn_2_GeO_4_ and ZnS are schematically displayed in Figure S3, Supporting Information, and a theoretical void space of 15.42 vol% is induced based on the volume of ZnS. To measure the carbon content of ZnS@C nanorods, thermogravimetric analysis was carried out from 40 to 700 °C in air condition (Figure 1g). According to the final product ZnO in air condition, the carbon content was determined as 21.2 wt%, and such a considerable percentage promises a good electronic conductivity of ZnS@C nanorods. The porous structure was also evaluated by nitrogen desorption/adsorption isotherms, and further analyzed by Barrett‐Joyner‐Halenda (BJH) method (Figure 1e,f). Certainly, the ZnS@C nanorods have a high surface area of 157.33 m^2^ g^−1^ with an average pore radius of 37.9 nm. Such a porous structure of ZnS@C nanorods endows considerable void space for volume expansion.

**Figure 2:**
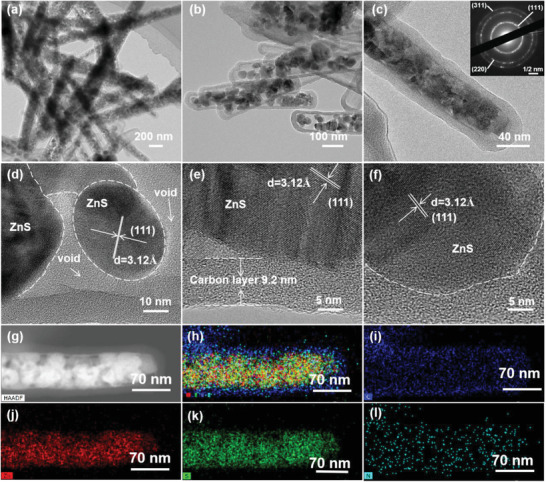
a–c) TEM images indicating the yolk of ZnS nanoparticles are fully encapsulated by the carbon nanotube, the insert of c is SAED patterns of a typical ZnS@C nanorod; d–f) HRTEM results showing the embedded ZnS nanocrystallites and carbon shell; HAADF‐STEM (g) and EDX‐mapping images (h–l) for a single ZnS@C nanorod: h) comprehensive element distribution of i) C element, j) Zn element, K) S element, and l) N element.

The detailed microstructure of these ZnS@C nanorods was deeply discovered by transmission electron microscopy (TEM). As exhibited in **Figure** **2**a, ZnS@C particles all display a nanorod structure with a diameter of 100–250 nm. The higher magnification TEM image directly proves the uniform encapsulation of ZnS nanoparticles in PDA‐derived carbon nanotube (Figure 2b,c). Separately, TEM images of pure PDA‐derived carbon nanotube are exhibited in Figure S4, Supporting Information, revealing a hexagonal nanotube structure that is inherited by the hexagonal Zn_2_GeO_4_ nanorods. Moreover, the high‐resolution TEM (HRTEM) images indicate the thickness of the carbon layer was ≈9.2 nm, and the mainly lattice fringes are corresponding to the (111) crystalline plane of cubic ZnS (Figure 2d–f). Figure 2g displays the high‐angle annular dark‐field (HAADF) TEM signal, which in‐depth verifies that ZnS particles are completely encapsulated in the hexagonal carbon nanotubes. To unveil the element distribution of porous ZnS@C rods, energy‐dispersive X‐ray (EDX) element mapping was also analyzed. All signals of Zn, S, N, C present as nanorod shape, and the signals of Zn, S elements overlap well which is confined in that of C, N elements (Figure 2h–l).

**Figure 3:**
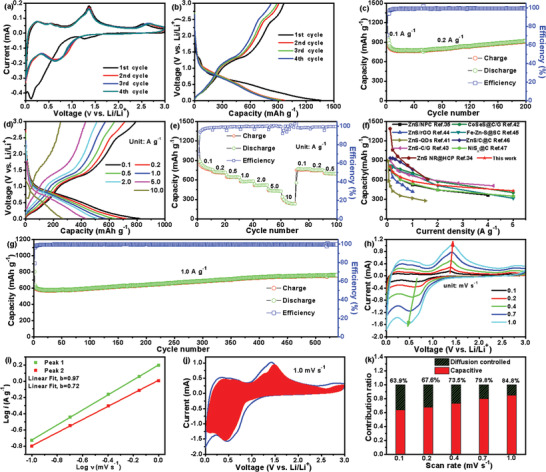
The detailed Li‐ion storage properties of yolk–shell ZnS@C nanorods: a) CV curves; b) discharge/charge profiles; c) cycle capability at 0.2 A g^−1^; discharge/charge curves (d) from 0.1 to 10 A g^−1^ and corresponding rate performances (e); f) the rate capability comparison of ZnS@C nanorods with recently published sulfide anodes; g) long‐cycle capability at 1.0 A g^−1^; h) CV curves of ZnS@C anode increased from 0.1 mV s^−1^ to 1.0 mV s^−1^; i) linear fitting graphs of peak current and sweep rate at anodic/cathodic peaks ≈1.3V/0.6 V; j) the red shaded region displays pseudocapacitive contribution at 1.0 mV s^−1^; k) the normalized contribution ratios of pseudocapacitive at different sweep rates.

To demonstrate the Li‐ion storage properties of this porous ZnS@C anode, half‐cells (coin type, CR2016) were assembled, and the galvanostatic discharge/charge and cyclic voltammetry tests were measured. As depicted in **Figure** **3**a, the initial cyclic voltammetry (CV) curves of ZnS@C show a different initial cathodic process that may be ascribed to the consumption of electrolytes to form the solid electrolyte interface (SEI). The following 2nd to 4th CV curves overlap well with each other indicating the highly electrochemical reversibility of these ZnS@C nanorods. It is worth noting that the cathodic peaks around 0.7 V and 0.01V are attributed to the conversion process transforming the ZnS to Zn/Li_2_S and further alloying process reducing the Zn to LiZn, respectively. Also, the initial four voltage‐capacity profiles in Figure 3b display discharge/charge platforms at about 0.6/1.3V, which are in accord with the cathodic/anodic peaks located in CV profiles. Furthermore, this ZnS@C achieves an initial Coulombic efficiency (ICE) of 70.7% accompanied by a discharge/charge capacity of 1358/960 mAh g^−1^. Figure 3c displays that the porous ZnS@C anode possesses 920 mAh g^−1^ after 200 cycles at 0.2 A g^−1^ and the cycling property is quite more stable than that of pure ZnS (only 540 mAh g^−1^ after 200 cycles at 0.1 A g^−1^, Figure S5, Supporting Information). Figure 3d shows the voltage‐capacity profiles of this ZnS@C anode at different charge rates from 0.1 to 10 A g^−1^ acquiring a capacity of 806, 720.9, 643.7, 576.1, 518.1, 431.5, 267.5 mAh g^−1^, respectively. There is almost no polarization below 2.0 A g^−1^, suggesting this yolk–shell structure endows its superior diffusion ability of Li^+^/electrons. The corresponding rate performance (Figure 3e) exhibits high stability at each discharge/charge rate directly proving excellent rate capability. Also compared with previously reported ZnS/ZnO‐based electrodes (Figure 3f), these ZnS@C yolk–shell nanorods show obvious advantages in the rate capability.^[^ ^34^, ^35^, ^41^, ^42^, ^43^, ^44^, ^45^, ^46^, ^47^ ^]^ Certainly, the long cycling performance and corresponding capacity‐voltage curves were carried out at 1.0 A g^−1^ (Figure 3g and Figure S6, Supporting Information), and they can still possess a high capacity of 710 mAh g^−1^ after 540 cycles. As for a large current density of 2.0 A g^−1^ (Figure S7, Supporting Information), ZnS@ nanorods also achieve 570 mAh g^−1^ over 800 cycles. The electrochemical performance of pure carbon nanotube was also measured as shown in Figure S8, Supporting Information. Due to the limited fraction of the content of this carbon nanotube, it only contributes a small proportion of capacity to the total ZnS@C anode. Especially, the electrochemical performance LiFePO_4_//ZnS@C full‐cell at 0.16 A g^−1^ (Figure S9, Supporting Information) was also measured, which obtained a stable capacity of 530 mAh g^−1^ over 50 cycles. To gain in‐depth insight into this porous ZnS@C anode achieved, CV curves of ZnS@C//Li half‐cell at different scan rates were carried out to measure the kinetic properties. As depicted in Figure 3h, CV curves increase from 0.1 mV S^−1^ to 1.0 mV S^−1^ with a similar shape and exhibit intrinsic cathodic/anodic peaks at ≈0.6 V/1.3 V. Fitting the *v^1/2 ^* versus *I_p_* (Figure S10, Supporting Information), we could determine the fitted linear with a slope of about 0.138. The Li^+^ diffusion coefficient (D_Li+_) can be calculated as about 8.64 × 10^−9^ cm^−2^ s^−1^ through the following simplified Randles‐Sevcik equation: ^[^ ^48^, ^49^ ^]^

(1) $$I_{p}=2.69\times 10^{5}AC{D_{Li+}}^{1/2}n^{3/2}v^{1/2}$$

Based on the Dunn's empirical formula, the current response (*i)* at a given sweep rate (*V*) abides the relationship:^[^ ^54^, ^55^ ^]^

(2) $$i=av^{b}$$

Herein *a* and *b* are the experimental parameters. Given Dunn's empirical formula, the *b*‐value of ZnS@C nanorods can be determined by fitting straight‐line of log *i* versus log *v*. Figure 3i exhibits the fitted lines with slopes of 0.72/0.97 representing *b*‐values of the cathodic/anodic peaks. All the calculated *b*‐values of this yolk–shell ZnS@C anode are both over 0.5, revealing that the lithiation/delithiation procedure is occupied with pseudocapacitive behavior. Furthermore, at a stated voltage, the diffusion‐controlled part (*k*_1_ *v* ^1/2^) and a pseudocapacitive fraction (*k*_2_ *v*) can be quantified abiding the following equation:^[^ ^50^, ^51^ ^]^

(3) $$i\left(V\right)=k_{1}v^{1/2}+k_{2}v$$

The Equation (3) can be reformulated as:

(4) $$i\left(V\right)/v^{1/2}=k_{1}+k_{2}v^{1/2}$$

Accordingly, the *k_2_*‐value can be easily acquired by fitting *i(V)*/*v* ^1/2^ versus *v* ^1/2^, and the pseudocapacitive fraction (*k_2_ν*) can be easily quantified.^[^ ^50^, ^51^ ^]^ Evidently, the yolk–shell ZnS@C anode equips an impressive pseudocapacitive contribution ratio of 63.9%, 67.6%, 73.5%, 79.8%, and 84.8% at 0.1, 0.2, 0.4, 0.7 to 1.0 mV s^−1^ (Figure 3j,k), respectively. At high scan rates, the ZnS@C possesses a high proportion of pseudocapacitive contribution illustrating that the Li‐ions storage procedure is occupied by superficial and interfacial storage behaviors. These yolk–shell ZnS@C nanorods, owing to their outer protective shell and unique interior porous space are capable of alleviating the volume change and preventing the aggregation of active ZnS, thus generating superior battery performance and good structure stability.

**Figure 4:**
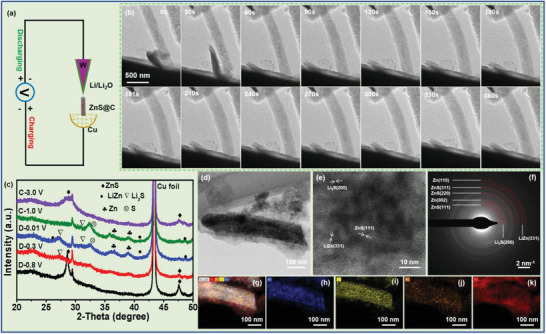
a) Schematic diagram of the in situ TEM; b) the in situ TEM images of a single ZnS@C nanorod at different discharge/charge states; c) the ex situ XRD of ZnS@C anode during the initial lithiation/delithiation process; d–k) the ex situ TEM characterization of ZnS@C anode at the state of discharge to 0.01V: d) TEM, e) HRTEM, f) SEAD and g–k) EDX‐mapping images of ZnS@C nanorod: g) comprehensive element distribution of h) Zn, i) S, j) N and k) C.

To in‐depth reveal the lithiation/delithiation mechanism and structural stability of this porous ZnS@C anode, in situ TEM and ex situ XRD characterizations were performed (**Figure** **4**). Figure 4a depicts the schematic diagram of the in situ TEM measurement, in which the W needle is dipped in Li/Li_2_O and Cu film adsorbs ZnS@C nanorods is used as the electrode with an external electric field. Figure 4b displays a single ZnS@C nanorod during the lithiation/delithiation process. The ZnS@C nanorod undergoes apparent expansion the diameter increases from 258 nm to 300 nm with the charge time from 0 to 180 s. During delithiation process, the diameter of ZnS@C nanorod shrinks; the diameter decreases from 300 nm to 285 nm with the discharge time from 181 to 361 s. It is worth noting that the carbon nanotube shell remains intact without breaking, meaning that the carbon nanotube is competent for tolerating the volume expansion during the lithiation/delithiation process. To provide more evidence to confirm the lithiation/delithiation mechanism, ex situ XRD and TEM were conducted to confirm new phases at the partly/fully lithiation states (Figure 4c–k). Both LiZn and Li_2_S phases can be found in Figures 4c and 4f, which further confirms the lithiation process of ZnS@C. Figure 4d shows TEM image of ZnS@C nanorod at a thorough lithiation state (0.01 V), revealing ZnS nanoparticles are still packaged in the carbon nanotube framework. HRTEM result (Figure 4e) of ZnS at fully lithiation state emerges various lattice fringes. Especially, the lattice spacings of 3.12 Å, 2.86 Å, 1.88 Å are ascribed to the (111) plane of ZnS, the (200) plane of Li_2_S and the (331) plane of LiZn, respectively.^52,53^ The corresponding EDX‐mapping signals of Zn, S, N, and C elements (Figure 4g‐k) are in keeping with nanorod shape, confirming the co‐existence of these elements. Ex situ XRD results at the full‐charged state (3.0 V) of ZnS@C exhibit the peaks of Li_2_S and LiZn fade away, suggesting Li_2_S and LiZn come back to ZnS during the delithiation process. On account of the above analysis, the lithiation/delithiation mechanism of cubic ZnS abides the following equations^[^ ^52^, ^53^ ^]^

(5) $$\mathrm{ZnS}+2Li^{+}+2e^{-}↔Li_{2}S+\mathrm{Zn}$$

(6) $$\mathrm{Zn}+Li^{+}+e^{-}↔\mathrm{LiZn}$$

**Figure 5:**
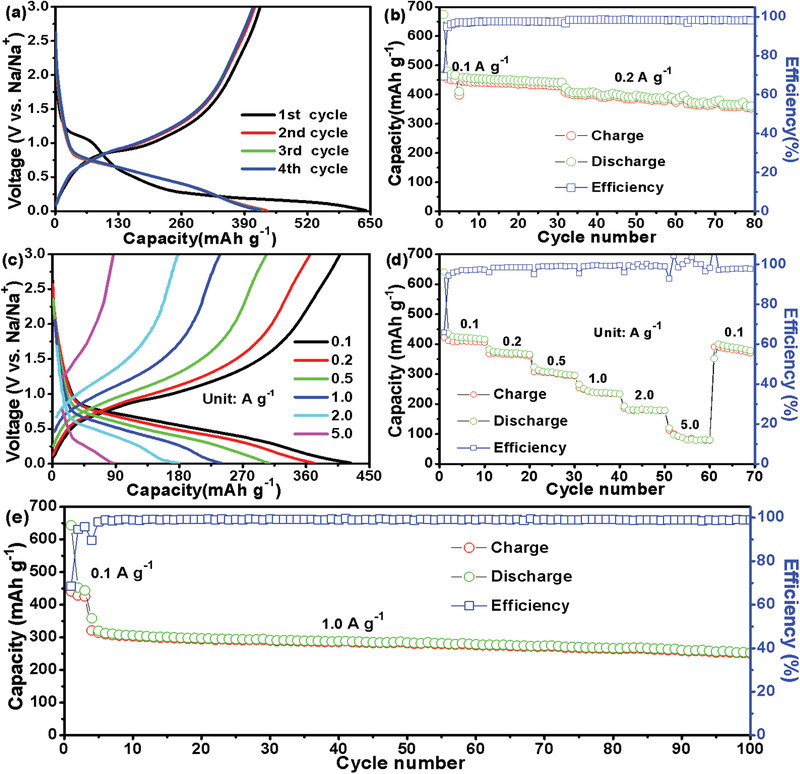
The detailed electrochemical performances of yolk–shell ZnS@C for SIBs: a) the initial four capacity‐voltage curves; b) cycle performance from 0.1 to 0.2 A g^−1^; c,d) discharge/charge curves (c) and rate performance (d) from 0.1 to 5.0 A g^−1^; e) long cycle properties at a high rate of 1.0 A g^−1^.

To reveal the Na‐ions storage performance, half‐cells were also assembled and measured. **Figure** **5**a shows the initial four voltage‐capacity profiles displaying a discharge/charge platform at about 0.55/1.0 V. Furthermore, this ZnS@C attains a high ICE of 65.94% along with a high discharge/charge capacity of 640.6/422.4 mAh g^−1^. Figure 5b depicts the cycling properties of ZnS@C from 0.1 to 0.2 A g^−1^ with a capacity of 360.8 mAh g^−1^ after 80 cycles. Figure 5c displays capacity‐voltage curves of yolk–shell ZnS@C nanorods at different charge/discharge rates (from 0.1, 0.2, 0.5, 1.0, 2.0 to 5.0 A g^−1^), in which the larger polarization than that of LIBs could be attributed to the larger radius of Na^+^ than Li^+^.^[^ ^54^ ^]^ Figure 5d reveals this ZnS@C attains a capacity of 410.3, 365.5, 303.9, 236.9, 180.6, 81.4 mAh g^−1^ from 0.1, 0.2, 0.5, 1.0, 2.0 to 5.0 A g^−1^, respectively. Certainly, the long cycling property was carried out at 1.0 A g^−1^ for 100 cycles, and this unique yolk–shell ZnS@C electrode possesses a capacity of 250.5 mAh g^−1^ (Figure 5e).

**Figure 6:**
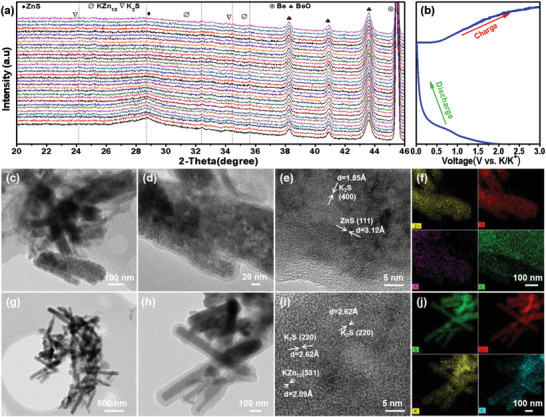
In situ XRD results (a) and corresponding discharge/charge profiles (b) of ZnS@C nanorods for KIBs; TEM results of ZnS@C at discharged state (0.3 V): TEM (c,d), HRTEM (e) images and corresponding EDX elemental mapping results (f); TEM results of ZnS@C at discharged state (0.01 V): TEM (g,h), HRTEM (e) images and corresponding EDX elemental mapping results (j).

With these yolk–shell ZnS@C, the corresponding KIBs were also assembled. To reveal the detailed reaction mechanism of ZnS@C during the K^+^ extraction/insertion, in situ XRD was firstly performed. As shown in **Figure** **6**a,b, the XRD peaks become weaker and then disappear, and weak peaks of KZn_13_ and K_2_S emerge during the initial insertion of K^+^.^[^ ^55^, ^56^, ^57^, ^58^, ^59^ ^]^ To further confirm the reaction mechanism acquired from in situ XRD, ex situ TEM was conducted, proving the KZn_13_ and K_2_S coexist at the partly/fully discharge state (0.3/0.01V). Figure 6c,d display TEM images of ZnS@C nanorods discharged to 0.3 V, which reveal that ZnS particles are still confined in the carbon nanotube. HRTEM image (Figure 6e) of ZnS at partly discharged state (0.3 V) shows various lattice fringes. Especially, the lattice spacings of 3.12 Å and 1.85 Å can be assigned to the (111) plane of cubic ZnS and the (400) plane of K_2_S, respectively. Furthermore, the EDX‐mapping signals of Zn, S, K, and C elements (Figure 6f) are present as nanorod shape, revealing the co‐existence of these elements. At the fully charged state, the ZnS@C (Figure 6g,h) still preserves the nanorod structure and the voids were filled by the expanded active materials which are originated from the transformation of ZnS into K_2_S and KZn_13_.^[^ ^55^, ^58^ ^]^ Especially, the lattice spacings of 2.09 Å and 2.62 Å (Figure 6i) can be indexed to the (531) plane of KZn_13_ and the (220) plane of K_2_S, respectively. The corresponding EDX‐mapping results (Figure 6j) of ZnS@C nanorods discharged to 0.01 V also appear as nanorod shape confirming the co‐existence and distribution of Zn, S, K, and C elements. On account of the above analysis results, the actual potassium/depotassium mechanism of cubic ZnS can be summarized as follows: ^[^ ^55^, ^56^, ^57^, ^58^, ^59^ ^]^

(7) $$\mathrm{ZnS}+2K^{+}+2e^{-}↔K_{2}S+\mathrm{Zn}$$

(8) $$13\mathrm{Zn}+K^{+}+e^{-}↔\mathrm{KZ}n_{13}$$

**Figure 7:**
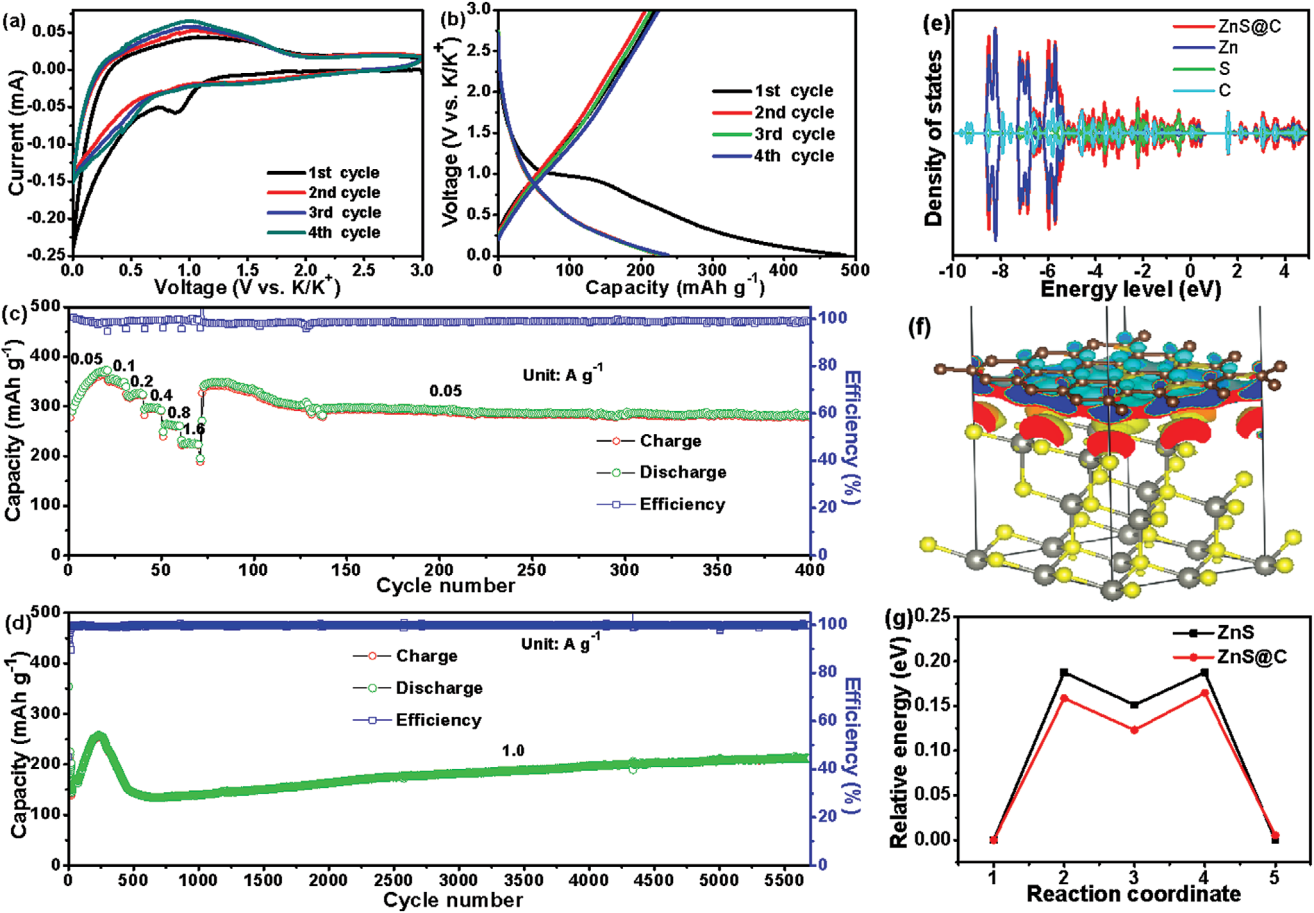
The electrochemical properties of yolk–shell ZnS@C rods for KIBs: a) CV curves; b) the discharge/charge curves at 0.1 A g^−1^; c) rate performances from 0.05 to 1.6 A g^−1^and return to 0.05 A g^−1^; d) ultra‐long cycle properties at 1.0 A g^−1^; e) the total density of states and projected density of states of ZnS/C interface; f) the charge density difference of ZnS/C interface, where blue indicates charge depletion and yellow represents charge accumulation; g) diffusion barrier energy (*_Δ_E*) of K^+^ along the optimized (111) plane in the bulk ZnS and ZnS@C nanorods.

To comprehensively evaluate the alkali ions storage performance of the ZnS@C nanorods, K‐ions batteries (coin type, CR2025) were assembled and their corresponding electrochemical performances were measured. As presented in **Figure** **7**a, the first four CV results of ZnS@C anode show a different initial cathodic process that may be ascribed to the consumption of electrolytes to form the SEI. The following 2nd to 4th CV curves are overlapped well with each other, indicating the high electrochemical reversibility of these ZnS@C nanorods. Also, the initial four voltage‐capacity profiles at 0.1 A g^−1^(Figure 7b) are consistent with the CV results. Furthermore, this ZnS@C acquires a discharge/charge capacity of 485.5/219.4 mAh g^−1^, with an ICE of 45.2%. Figure 7c shows the rate performance recorded at different charge rates from 0.05, 0.1, 0.2, 0.4, 0.8 to 1.6 A g^−1^ with a capacity of 359.8, 347.7, 320.6, 295.3, 261.7, 224.5 mAh g^−1^, respectively. The stable cycling performance at each discharge/charge rate directly proves excellent rate capability. Certainly, the long cycling performance (Figure 7d) and capacity‐voltage profiles (Figure S11, Supporting Information) of the porous ZnS@C anode possesses a high capacity of 211.1 mAh g^−1^ at 1.0 A g^−1^ even after 5700 cycles_._ To in‐depth reveal the superior K‐ion storage cycling performance, the partial density of states (PDOS) of C, Zn, S, and ZnS@C was calculated with the bonding valence‐electron states, and their electron structures for Zn, S, and C atoms (*p* states of C, S and *d* state of Zn) are depicted in Figure 7e.^[^ ^60^, ^61^ ^]^ There appears a slight enhance of electronic states for ZnS@C near the Fermi level when compared to that of their single components (Zn, S, and C). This result demonstrates that the introduction of pyrolytic carbon does not significantly change the electronic structure of ZnS.^[^ ^60^, ^61^ ^]^ Furthermore, the electrons on the graphene are transferred to the ZnS surface, and there are obvious negative electron layers (blue, electron density decrease) and positron‐electron layers (yellow, electron density increase).^[^ ^60^, ^61^ ^]^ Bader charges results show that there is an increased charge transfer from C to ZnS at the ZnS/C interface (0.29 electrons/atom). In addition, the exact diffusion barriers of ZnS@C and ZnS along the (111) plane were also calculated and the detailed diffusion channels were provided in Figure S12, Supporting Information. As displayed in Figure 7g, the ZnS@C with a diffusion barrier of 0.16 eV is lower than that of pure ZnS (0.19 eV). The density functional theory (DFT) calculation results indicate that the coupling between ZnS and C seriously influences the diffusion barrier of K^+^, thereby improving the overall storage performance of the porous ZnS@C. It is believed that the carbon nanotube suppresses the aggregation of ZnS particles, which effectually accelerated the K^+^ diffusion rate in the ZnS@C. To reveal the difference between LIBs, SIBs, and KIBs, the EIS spectra of ZnS@C for LIBs, SIBs, KIBs and the relationship plots between *Z* and *ω* ^1/2^ derived from the low‐frequency region of the corresponding EIS spectra were added in Figure S13, Supporting Information. The corresponding diffusion coefficient of Li^+^, Na^+^, and K^+^ in ZnS@C anode could be determined as 9.0 × 10^−13^, 4.2 × 10^−14^, and 1.06 × 10^−15^ S cm^−2^, respectively. This phenomenon could be ascribed to the larger radius of Na^+^ and K^+^ (versus Li^+^) causing a larger diffusional resistance.

## 3 Conclusion

In summary, a yolk–shell ZnS@C electrode with considerable void space is obtained via a self‐sacrificial template route. Such yolk–shell ZnS@C electrode exhibits superior rate performance and cycling performance (740 mAh g^−1^ after 540 cycles at 1.0 A g^−1^) for LIBs. As a KIBs anode, the current ZnS@C achieves an ultra‐long lifespan delivering 211.1 mAh g^−1^ at 1.0 A g^−1^ after 5700 cycles. The kinetic analysis reveals that these yolk–shell ZnS@C nanorods with considerable pseudocapacitive contribution benefit the fast lithiation/delithiation process. Detailed in situ TEM and ex situ XRD tests for LIBs, in situ XRD and ex situ TEM analysis for KIBs indicate that this yolk–shell ZnS@C anode show a typical reversible conversion reaction mechanism accomplished by alloying processes. The superior alkali‐ions storage performance can be ascribed to that the carbon encapsulated structure effectively moderates integral electronic conductivity and ensures rapid alkali‐ions/electrons transporting. Furthermore, the rationally designed structure of ZnS@C nanorods endows enough void space to mitigate volume stress during the alkali‐ions insertion/extraction. This self‐sacrificial template strategy will open an avenue for designing porous materials and broaden an application field not limited to alkali‐ion batteries, supercapacitors, and catalysis.

## Conflict of Interest

The authors declare no conflict of interest.

## Supporting information

Supporting Information

## Data Availability

The data that support the findings of this study are available from the corresponding author upon reasonable request.
