# Supplementary material for: Self‐Sacrifice Template Construction of Uniform Yolk–Shell ZnS@C for Superior Alkali‐Ion Storage
Source: Adv Sci (Weinh). 2022 Mar 15;9(14):2200247. doi: 10.1002/advs.202200247 (PMC9108611; doi:10.1002/advs.202200247)
Supplement: Supplementary file 1 — Supporting Information [file ADVS-9-2200247-s001.pdf]

ADVANCED SCIENCE
Open Access

Supporting Information

for *Adv. Sci.*, DOI 10.1002/advs.202200247

Self-Sacrifice Template Construction of Uniform Yolk–Shell ZnS@C for Superior Alkali-Ion Storage

*Xijun Xu, Fangkun Li, Dechao Zhang, Zhengbo Liu, Shiyong Zuo, Zhiyuan Zeng and Jun Liu**

Supporting Information

Self-Sacrifice Template Construction of Uniform Yolk-Shell ZnS@C for Superior Alkali-Ion Storage

*Xijun Xu, Fangkun Li, Dechao Zhang, Zhengbo Liu, Shiyong Zuo, Zhiyuan Zeng, Jun Liu**

Experimental Section/Methods

Synthesis of yolk-shell ZnS@C nanorods

The precursor of Zn_2GeO_4 was prepared *via* a conventional hydrothermal route. 0.52 g GeO_2, 1.1 g $Zn(CHCOO)_2{\cdot}2H_2O$ and 0.364 g cetyl trimethyl ammonium bromide (CTAB) were poured into 60 ml ultrapure water and the pH was adjusted 10 with 2 M NaOH. Subsequently, the white mixed solution was transferred into a Teflon reactor at 140 °C for one day. The Zn_2GeO_4 was acquired by washing the precipitate with ethanol and ultrapure water and drying in vacuum condition. Afterward, 0.168 g tris(hydroxymethyl)aminomethane and 0.12 g obtained Zn_2GeO_4 were ultrasonically dispersed in 150 mL ultrapure water/ethanol (1:1 in volume). Then, 0.06 g dopamine was added with vigorous stirring for one day. Then the brown suspension was washed and dried in vacuum condition. The collected sample was further annealed at 500 °C for 3 h with Ar flowing to prepare Zn_2GeO_4@C nanorods. Finally, 1.56 g $Na_2S{\cdot}9H_2O$ and 0.2 g Zn_2GeO_4@C nanorods were dispersed in 60 mL water with stirring and then transferred into a 100 mL Teflon reactor at 200 ºC for 12 h. Finally, the yolk-shell ZnS@C nanorods were obtained by centrifuging with ultrapure water and ethanol and then drying in a vacuum oven.

Materials characterization

The morphology of these samples was conducted on a scanning electron microscope (SEM, Zeiss Gemini DSM 982). The crystalline structure information was recorded on an X-ray diffraction (XRD) device (PANalytical Empyrean) at 45 mA and 40 kV With Cu Kα radiation. The microstructure and structure evolution of ZnS@C were revealed by JEOL 2100, 2100F, and Talos F200X transmission electron microscope (TEM). A Quadrachrome adsorption instrument was applied to analyze the surface area of ZnS@C and the corresponding pore condition was revealed by a Barrett-Joyner-Halenda (BJH) method. The carbon content of ZnS@C nanorods was conducted on a thermogravimetric analysis (TGA) system (TG 209 F3 Tarsus) with an oxygen atmosphere from 40 °C to 700 °C with 10 °C min^{-1}.

Electrochemical tests

The preparation for the electrode was mixing 70wt% ZnS@C, 15wt% Super P, 15wt% sodium alginate, and appropriate water with homogeneously stirring. Next, the prepared slurry was painted on the Cu foil and at 90 °C for overnight under vacuum conditions. Finally, the working electrodes were cut into 12 mm disks with mass loading of 0.7~1.1 mg cm^{-2}. The assembling process for half-/full-cells occurred in an Ar-filled glove box (both H_2O and O_2 lower than 0.1 ppm). For Li-ion batteries, 1 M $LiPF_6$ dissolved in diethyl carbonate/ethylene carbonate (DEC/EC, 1: 2 by volume) containing 10 wt% fluoroethylene carbonate (FEC) was used as the electrolyte. The purchased Li-disks and monolayer polypropylene (Celgard 2400) were adopted as counter electrodes and separators, respectively. The $LiFePO_4$ cathode was purchased from Guangdong Canrd New Energy Technology Co., Ltd. with a mass loading of ~11.5 mg cm^{-2}. For sodium-ion batteries (SIBs), 1 M $NaClO_4$ dissolved in diethyl carbonate/ethylene carbonate (DEC/EC, 1: 1 by vol) containing 5 wt% FEC was used as the electrolyte. Na disks and glass fiber (Whatman GF/D) were adopted as the counter electrodes and separators, respectively. For potassium ion batteries, a 3 M potassium bis-fluoromethane-sulfonyl imide (KTFSI) in diglyme (DME) was used as the electrolyte. Freshly made K disks and glass fiber (Whatman GF/D) were adopted as the counter electrodes and separators, respectively. Cyclic voltammetry (CV) profiles were collected on an electrochemical workstation (Gamry Interface 1000) at different scan rates in the region of 0.01~3.0 V. The discharge/charge tests were recorded in the windows of 0.01~3.0 V using a multichannel battery testing system (Land 2001A).

Computational details

All theoretical spins are simulated on the Vienna Ab-initio Simulation Package (VASP).[S1] The electron-electron correlation interactions and exchange of ZnS@C were revealed by using the generalized gradient approximation (GGA) with the Perdew-Burke-Emzerhof (PBE) functional form.[S2] The valence electron interactions and electronic structure were optimized by the projector augmented-wave (PAW) methods.[S3] The plane-wave basis function with kinetic cut-off energy is set as 550 eV. Monkhorst-Pack meshes with the size of $5 \times 5 \times 1$ were applied to sample the surface Brillouin zone.[S4] The ground-state atomic geometries were optimized by relaxing the force <0.02 eV/Å and the convergence criteria for energy was 1.0×10^{-5} eV/cell. The electronic structure and total energy calculation were using the gaussian method. To better describe the interactions between molecules, Van der Waal interactions are included and analyzed by the DFT-D3 method of Grimme.[S5] The transition

states during the reaction pathway were determined, using the climbing-image nudged elastic band (CI-NEB) method with convergence criteria of force < 0.05 eV/Å.[S6, S7]

For the ZnS@C nanorods, The weight loss in the region of 0~350 ℃ can be attributed to the absorbed water and functional group of carbon nanotube evaporation.[S8] The weight loss in the region of 350~550 ℃ was associated with the carbon nanotube combustion.[S8] The weight loss in the region of 350~550 ℃ was related to the conversion of ZnS into ZnO.[S8] So, there are three processes during the pyrolysis at air atmosphere as follows:[S8]

$$H_2O\ (l) \rightarrow H_2O\ (g)\ \ (0\text{~}350\ ℃) \quad (S1)$$

$$C\ (s) + O_2\ (g) \rightarrow CO_2\ (g)\ \ (350\text{~}550\ ℃) \quad (S2)$$

$$ZnS\ (s) + O_2\ (g) \rightarrow ZnO(s) + SO_2\ (g)\ \ (550\text{~}700\ ℃) \quad (S3)$$

Based on the TGA measurement curves, the residual component is ZnO when they are heated to 700 ℃. According to the conservation of Zn element, the mass percentage of ZnS in the ZnS@C can be calculated as 78.8 wt%.

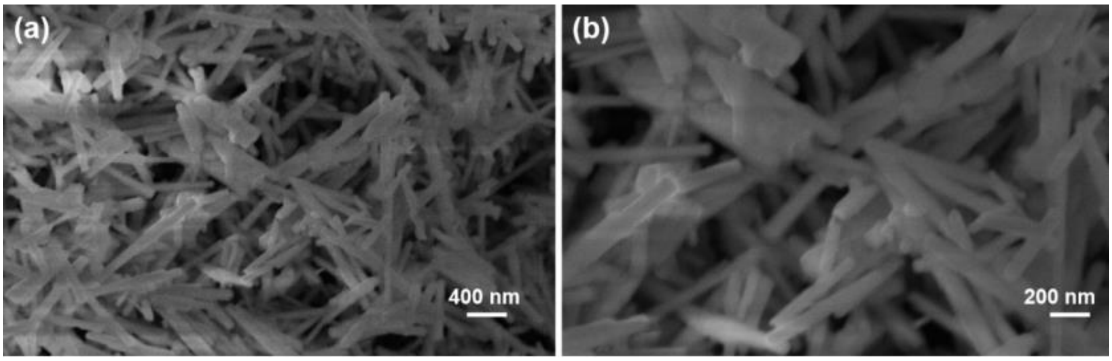

Figure S1.SEM images of the precursor of Zn_2GeO_4@C nanorods.

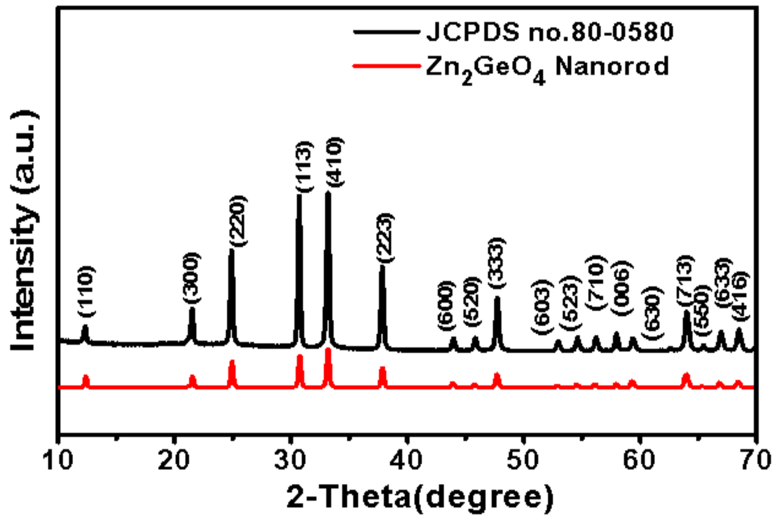

Figure S2. The typical XRD pattern of the precursor of Zn_2GeO_4.

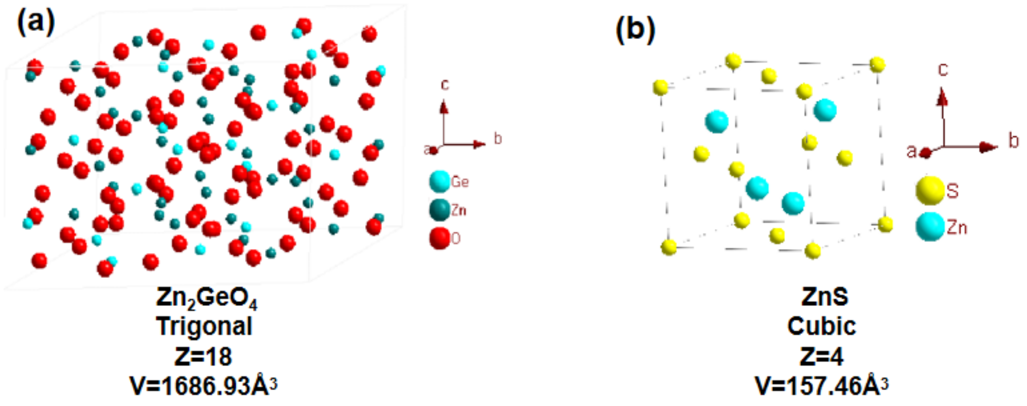

Figure S3. Schematic diagram of crystalline structures: (a) Zn_2GeO_4; (b) ZnS.

The detailed chemical reaction relevant to the material synthesis process of the ZnS@C nanorods under the alkaline hydrothermal conditions is shown as follows:

$$Zn_2GeO_4 + 2S^{2-} + H_2O \rightarrow ZnS + GeO_3^{2-} + 2OH^- \quad (S4)$$

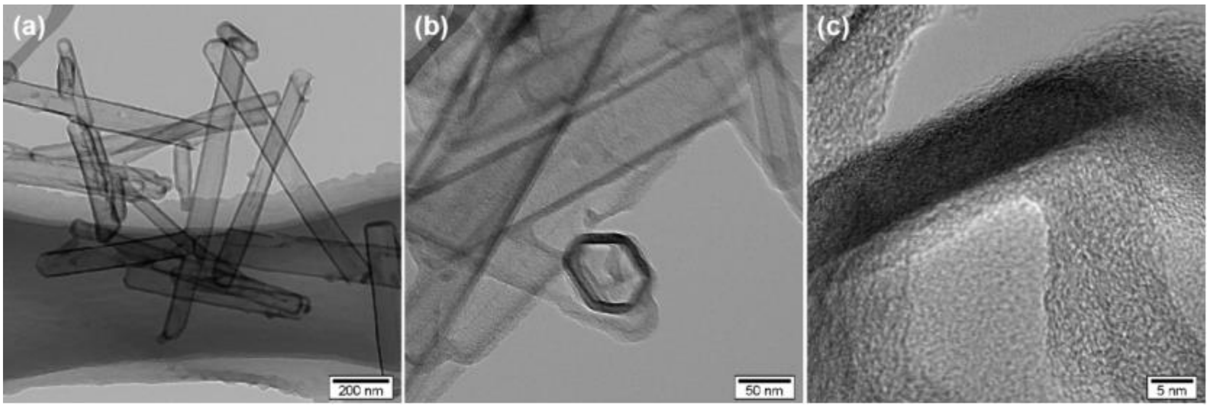

Figure S4. TEM (a,b) and HRTEM (c) images of PDA-derived carbon nanotubes by evaporating the Zn_2GeO_4 at 800 ºC for 6 h under 20%H_2/Ar atmosphere.

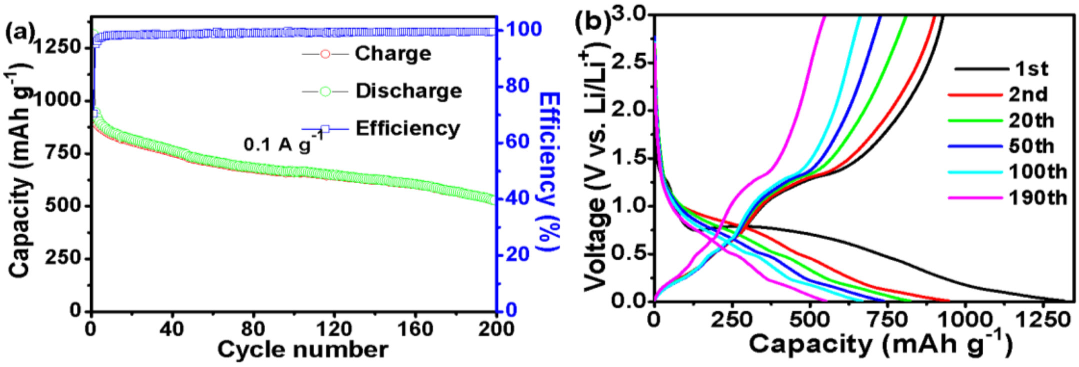

Figure S5. (a) The cycling properties of pure ZnS and (b) the corresponding voltage-capacity profiles.

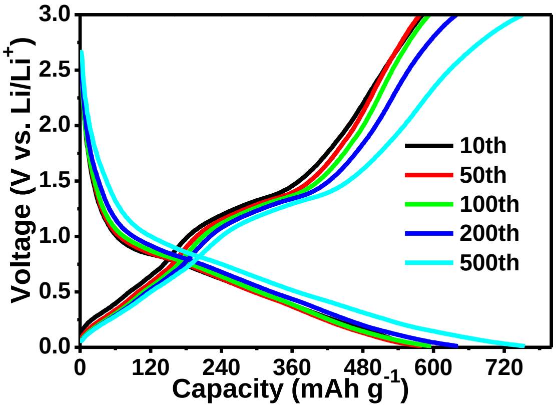

Figure S6. The corresponding voltage-capacity profiles at 10th, 50th, 100th, 200th, and 500th of ZnS@C for LIBs.

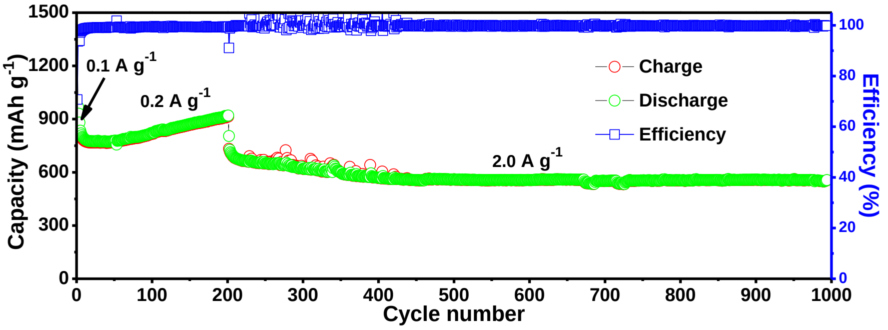

Figure S7. The long-cycle performance of ZnS@C at 2.0 A g^{-1} over 800 cycles.

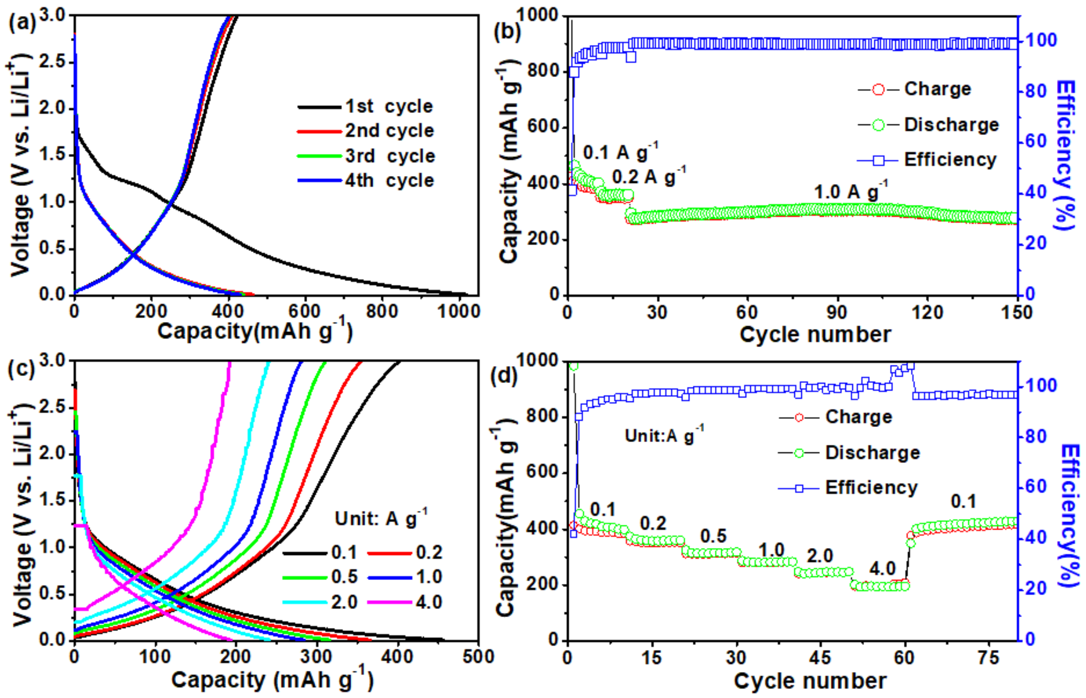

Figure S8. The electrochemical properties of carbon nanotube for LIBs: (a) the discharge/charge curves at 0.1 A g^{-1}; (b) long cycling properties at 1.0 A g^{-1}; (c) the charge/discharge profiles and (d) rate performances from 0.1 to 4.0 A g^{-1}.

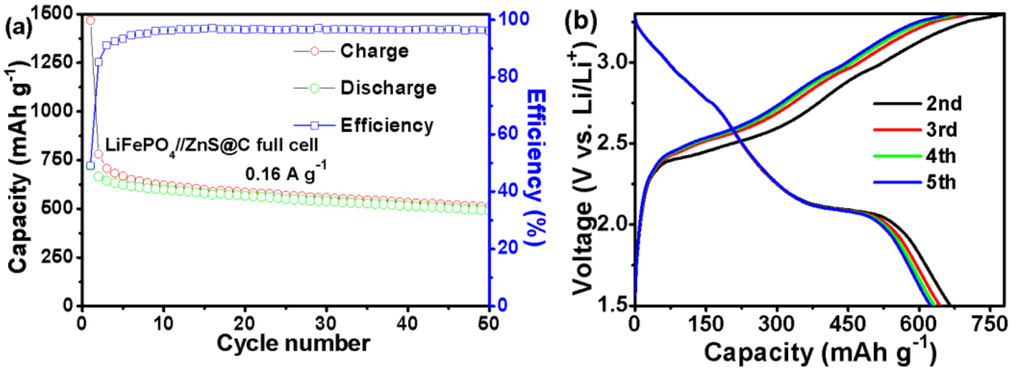

Figure S9. (a) The full cell performance of $LiFePO_4$//ZnS@C at 0.16 A g^{-1} over 50 cycles and (b) the corresponding voltage-capacity profiles.

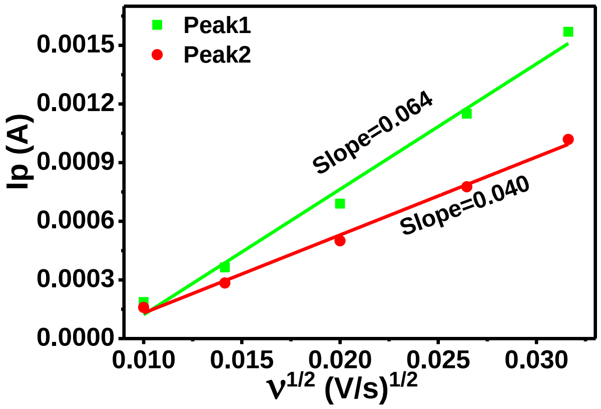

Figure S10. The relationship between the peak currents and sweep rates is associated with Li^+ insertion/extraction in ZnS@C nanorods.

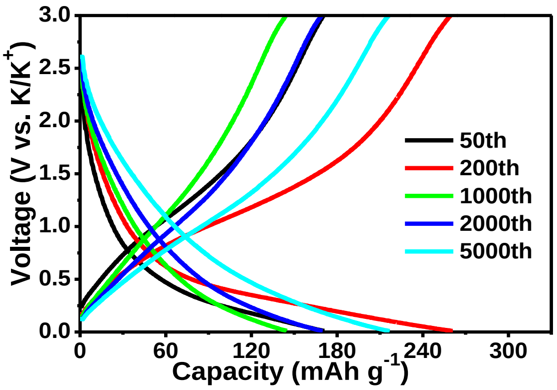

Figure S11. The corresponding voltage-capacity profiles at 50th, 200th, 1000th, 2000th, and 5000th of ZnS@C for KIBs.

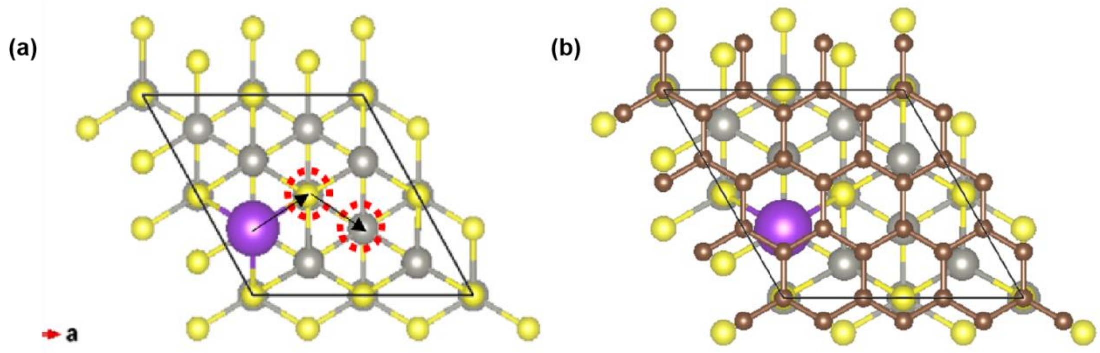

Figure S12. The calculated diffusion channels of ZnS (a) and ZnS@C (b) on the (111) plane.

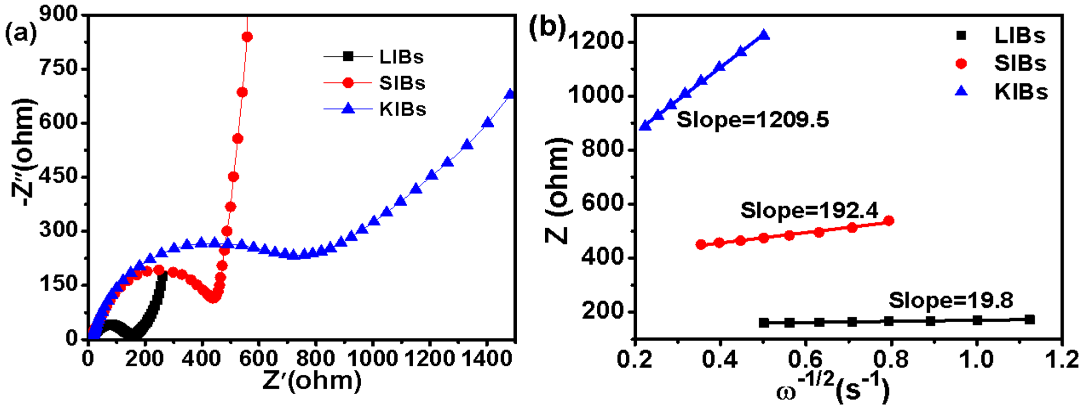

Figure S13. (a) Nyquist plots of ZnS@C for LIBs, SIBs, and KIBs; (f) the corresponding relationship plots between Z and $\omega^{1/2}$.

As displayed in Figure S13, the relationship plots between Z and $\omega^{1/2}$ can be derived from the low-frequency region of the corresponding EIS spectra. Moreover, the apparent diffusion coefficient of Li^+, Na^+, K^+ concerned with σ can be estimated from the low-frequency region according to the following equation:[S9, S10]

$$D = \frac{R^2T^2}{2A^2n^4F^4C^2\sigma^2} \quad \text{(S5)}$$

According to the equation and calculated σ value, the corresponding diffusion coefficient of Li^+, Na^+, K^+ in ZnS@C anode could be determined as 9.0×10^{-13}, 4.2×10^{-14}, and 1.06×10^{-15} S cm^{-2}, respectively.

Table S1. A comparison of cycling performance between the current yolk-shell ZnS@C and other Zn-based chalcogenide anodes for KIBs.

Anode	Current density (mA g^{-1})	Discharge capacity (mAh g^{-1})	Cycle number (cycles)	Voltage region (V)	Reference
3D ZnS@C	1000	230	2300	0.01-3.0	Ref. S11
ZnSe NP@NHC	100	132.9	1200	0.3-2.9	Ref. S12
ZnSe@PCNF	500	270	1000	0.01-3.0	Ref. S13
ZnSe/C nanocages	500	189	1000	0.01–2.5	Ref. S14
ZnSe@C nanorods	2000	204	100	0.01-3.0	Ref. S15
ZnSe@NDPC	100	262.8	200	0.01-3.0	Ref. S16
ZnS@C@RGO	500	208	300	0.01–2.5	Ref. S17
ZnS QDs-rGO	100	350.4	200	0.01-3.0	Ref. S18
**Yolk-shell ZnS@C**	**1000**	**211**	**5700**	**0.01-3.0**	**This work**

References

[S1] P. Bliichl, *Phys. Rev. B*, **1994**, *50*, 17953.

[S2] G. Kresse, D. Joubert, J. *Phys. Rev. B* **1999**, 59, 1758.

[S3] J. P. Perdew, K. Burke, M. Ernzerhof, *Phys. Rev. Lett.*, **1996**, *77*, 3865.

[S4] H. J. Monkhorst, J. D. Pack, *Phys. Rev. B*, **1976**, *16*, 1748–1749.

[S5] L. Wang, Y. Sun, K. Lee, D. West, Z. Chen, J. Zhao, S. Zhang, *Phys. Rev. B* **2010**, *82*, 161406.

[S6] G. Henkelman, B. P. Uberuaga, H. Jónsson, *J. Chem. Phys.* **2000**, *113*, 9901.

[S7] G. Henkelman, H. Jónsson, *J. Chem. Phys.* **2000**, *113*, 9978.

[S8] X. Wei, H. Yuan, H. Wang, R. Jiang, J. Lan, Y. Yu, X. Yang, *Mater. Chem. Front.* **2021**, *5*, 4712.

[S9] F. Wang, N.Zhang, X.Zhao, L. Wang, J. Zhang, T. Wang, F. Liu, Y. Liu, L. Z. Fan, *Adv. Sci.* 2019, 6, 1900649.

[S10] B. Wu, X. Yang, X. Jiang, Y. Zhang, H. Shu, P. Gao, L. Liu, X. Wang, *Adv. Funct. Mater.* **2018**, *28*, 1803392.

[S11] X. Xu, D. Zhang, Z. Wang, S. Zuo, J. Yuan, R. Hu, J. Liu, *ACS Appl. Mater. Interfaces* **2021**, *13*, 11007.

[S12] Y. He, L. Wang, C. Dong, C. Li, X. Ding, Y. Qian, L. Xu, *Energy Storage Mater.* **2019**, *23*, 35.

[S13] J. H. Na, Y. C. Kang, S.-K. Park, *Chem. Eng. J.* **2021**, *425*, 131651.

[S14] J. Chu, W. A. Wang, Q. Yu, C.-Y. Lao, L. Zhang, K. Xi, K. Han, L. Xing, L. Song, M. Wang, *J. Mater. Chem. A* **2020**, *8*, 779.

[S15] X. Xu, B. Mai, Z. Liu, S. Ji, R. Hu, L. Ouyang, J. Liu, M. Zhu, *Chem. Eng. J.* **2020**, *387*, 124061.

[S16] J. Y. Hu, T. Lu, Y. Zhang, Y. Sun, J. Liu, D. Wei, Z. Ju, Q. Zhuang, *Part. Part. Syst. Charact.* **2019**, *36*, 1900199.

[S17] Chu, W. A. Wang, J. Feng, C.-Y. Lao, K. Xi, L. Xing, K. Han, Q. Li, L. Song, P. Li, *ACS Nano* **2019**, *13*, 6906.

[S18] Y. Qi, Y. Yang, Q. Hou, K. Zhang, H. Zhao, H. Su, L. Zhou, X. Liu, C. Shen, K. Xie, *Chin. Chem. Lett.* **2021**, *32*, 1117.
